# Intra-abdominal pressure, vertebral column length, and spread of spinal anesthesia in parturients undergoing cesarean section: An observational study

**DOI:** 10.1371/journal.pone.0195137

**Published:** 2018-04-03

**Authors:** Ting-ting Ni, Ying Zhou, An-cui Yong, Lu Wang, Qing-he Zhou

**Affiliations:** 1 Department of Anesthesiology, Ningbo NO.7 Hospital, Ningbo, Zhejiang Province, China; 2 Department of Gynecology, Ningbo NO.7 Hospital, Ningbo, Zhejiang Province, China; 3 Department of Anesthesiology, the Second Affiliated Hospital, Jiaxing University, Jiaxing, Zhejiang Province, China; Massachusetts General Hospital, UNITED STATES

## Abstract

**Background:**

In parturients with increased physiologically intra-abdominal pressure (IAP) and a short stature, a greater cephalad spread of spinal anesthesia is often observed after a fixed amount of plain bupivacaine is administered. Therefore, we designed this prospective study to test whether IAP and vertebral column length (VCL) were predictors of spinal spread in parturients undergoing a cesarean section.

**Methods:**

A total of 113 parturients, all undergoing elective cesarean sections with single-shot spinal anesthesia, were enrolled. The L3-L4 interspace was entered, and 2 mL of 0.5% plain bupivacaine was injected into the subarachnoid space. Upon loss of temperature sensation at the T4 level, IAP was measured through a bladder catheter while the patient was in the supine position with a 10°left lateral tilt. Parturient demographic variables, including age, height, weight, IAP, and VCL were recorded. Linear regressions and multiple regressions were performed to analyze the relationships between parturient variables and the spread of spinal anesthesia.

**Results:**

A total of 109 parturients were included in the analysis. Linear regression analysis showed a significant univariate correlation of height, weight, body mass index (BMI), IAP, and VCL with cephalad spread (all *P*< 0.004). Multiple linear regression analysis showed that IAP and VCL were the key determinants of spinal spread (both *P* < 0.0001), where as exclusion of age, weight, and height did not change the result (all *P*> 0.209).

**Conclusions:**

Our data indicated that IAP and VCL were significant predictors of intrathecal spread of plain bupivacaine, and there was a positive association between IAP and abdominal girth in term parturients.

## Introduction

Regional administration of anesthesia using local anesthetics is the preferred anesthetic technique for cesarean delivery.[1] Plain bupivacaine is often used for spinal anesthesia.[2–4] However, the intrathecal spread of plain bupivacaine is highly unpredictable.[5] Many of the physiological changes that occur during pregnancy increase the effect of local anesthetics. Some previous studies reported that lumbosacral cerebrospinal fluid (CSF) volume and pressure were the primary determinants of spinal cephalad spread,[6, 7] but that information had little practical value because the measurement of CSF was inconvenient. Patient characteristics such as height, weight, and body mass index are frequently used to predict spinal anesthesia spread, but the results have been unsatisfactory.[8–10]

A recent study revealed that abdominal girth and vertebral column length (VCL)have a significant predictive value for the cephalad spread of spinal anesthesia in non-pregnant patients,[11] a result that was also found in term parturients.[12] Sugerman H, et al.[13]reported that abdominal girth was associated with intra-abdominal pressure (IAP) in non-pregnant patients. The relationship between abdominal girth and IAP in non-pregnant patients or in term parturients may be different because factors such as pregnancy or morbid obesity can cause different IAPs in patients with the same abdominal girth. Thus, we aimed to investigate whether IAP and VCL were significant predictors of spinal spread of plain bupivacaine and whether there was a positive association between IAP and abdominal girth in term parturients.

## Materials and methods

### Subjects

This study was approved by the Ethical Committee of Ningbo NO.7 Hospital on May 9^th^, 2017 and pre-registered at http://www.chictr.org.cn/index.aspx (ChiCTR-OON- 17011388). Informed consent was obtained from all participants. From May 10^th^ toJune20^th^2017, a total of 113parturients undergoing elective cesarean section under single-shot spinal anesthesia were enrolled in this prospective observational study. Patients with pre-eclampsia, diseases leading to peripheral edema or ascites, contraindications for spine anesthesia, a history of allergy to bupivacaine, or a history of spinal puncture failure were excluded, as were patients who needed additional intra-operative analgesia.

### Study protocol

All of the term parturients fasted for 8–10 h before the cesarean section. After the parturient entered the operating room and intravenous access was established, Ringer’s lactate 500 mL was preloaded, and standard monitoring was started. The parturient was placed on a horizontal operating table in the supine position, and during the end of expiration, the abdominal girth was measured at the level of the umbilicus. VCL was measured from the middle oftheC7 vertebra to the sacral hiatus while the parturient was sitting on the operating table with a straightened back. The C7 vertebra and sacral hiatus were both confirmed using ultrasound imaging. The L3-L4 interspace was selected as the location for puncture with a 25-gauge Quincke needle using a midline approach and a cephalad level. A total of 2mL of 0.5% plain bupivacaine was injected intrathecally over a period of 10 seconds when free flow of CSF was obtained. Parturients were then positioned in a 10° left-lateral tilt anda10° Trendelenburg position, which was confirmed using an angle measurement instrument (BOSCH, GAM220, Stuttgart, Germany).

The spinal spread was assessed in both midclavicular lines every minute using an 18-gauge needle for loss of pinprick sensation and using ice for loss of temperature sensation. When bilateral loss of cold sensation to the T4 dermatome level was achieved, surgery commenced, and the operating table was maintained at a left lateral tilt while being returned to a horizontal position. Time to achieve the T4 block level was defined as the time between the intrathecal injection and loss of temperature sensation at the T4 level. After establishing a T4 sensory block level, a transurethral catheter was inserted to drain the bladder and measure IAP in the supine position with a 10° left lateral tilt, as described by Chun et al.[14]

Spinal spread assessment continued every 2 minutes until the spinal spread remained unchanged for three consecutive assessments. The total number of intrathecal bupivacaine block segments between the fifth sacral vertebra and the segment of cephalad spread was recorded. General anesthesia was administered if spinal administration failed to produce a satisfactory level of surgical anesthesia.

All spinal anesthesia procedures and measurements were performed by the same attending physician, and the assessment of the spread of spinal anesthesia was completed by another anesthetist who was blind to the previous parturient’s measurements, such as age, height, weight, VCL, IAP, and abdominal girth. Bradycardia (defined as heart rate<50 beats/min) was treated with 0.5 mg of intravenous atropine. The systolic blood pressure baseline was measured three times before anesthesia, and the average was recorded (with an interval measurement time of 2min). Hypotension (defined as a decrease in systemic arterial pressure of >30% below baseline or<90 mmHg) was treated with 5mg of ephedrine administered intravenously.

### Measurements

Parturient demographic variables, including age, height, weight, length of gestation, gravidity, number of fetuses, parity, IAP, and VCL were recorded. We also recorded maximum sensory block level, including loss of temperature sensation and pinprick discrimination level, and neonatal weight.

### Statistical analysis

Five predictors of intrathecal spread were included in current study. If the desired statistical power level was 0.8, the probability level was 0.05, the anticipated effect size was 0.15, the predictors were 5, the minimum required sample size would be 92, and an additional 20% parturients were recruited to account for loss to follow-up. Statistical analysis was implemented with SPSS software (version 21.0, SPSS Inc., Chicago, IL, USA). A linear regression analysis was used to determine the correlations of the spinal anesthesia-induced loss of temperature sensation and pinprick discrimination with age, weight, height, IAP, and VCL. Stepwise selection was performed in order to determine which factors were the primary predictors during multiple regression analysis. R^2^ is the determination coefficient of multiple linear regression analysis. A *P* value < 0.05 was considered to be statistically significant.

## Results

**Fig 1:**
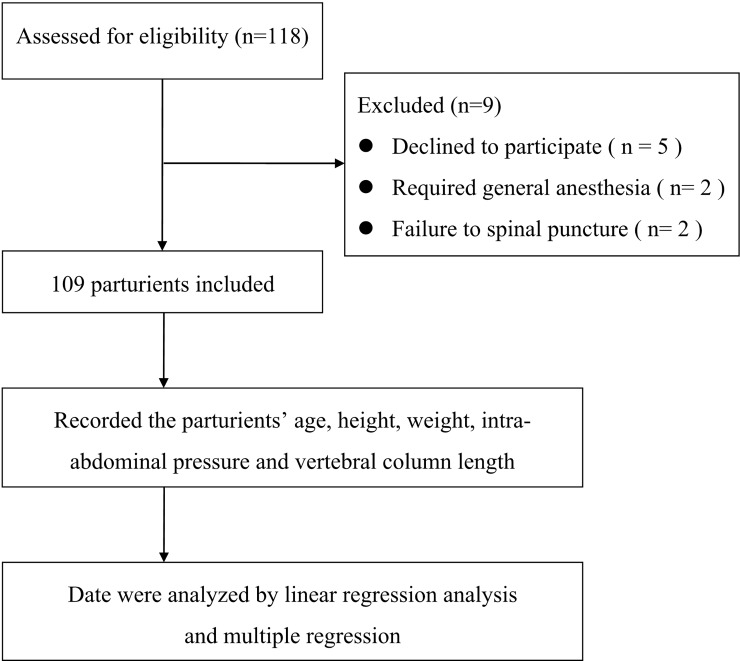
Flow diagram outlining the study procedure.

Four parturients were excluded from analysis: two of these required general anesthesia, and two had spinal puncture failure. The remaining 109 parturients completed the study according to the protocol and were included in the analysis (Fig 1). The characteristics of the parturients and neonates are presented in Table 1.

**Table 1:** Parturient characteristics and cephalad spread of spinal anesthesia (n = 109).

Age, y (mean ± SD)	28.0± 5.1
Height, cm (mean ± SD)	158.0±4.0
Weight, kg (mean ± SD)	70.8±8.4
IAP, mmHg (mean ± SD)	13.1±1.6
VCL, cm (mean± SD)	57.1±3.8
Abdominal girth, cm (mean ± SD)	100.9±6.4
BMI, kg/m^2^(mean ± SD)	28.2±3.0
Fetal biparietal diameter, cm (mean ± SD)	9.3±0.5
Gestation, weeks (median [interquartile range])	39.0 [38.0–40.0]
Gravidity (median [interquartile range])	2.0 [1.0–3.0]
Parity (median [interquartile range])	1.0 [0.0–1.0]
Singleton pregnancy (n (%))	103(94%)
Twin pregnancy (n(%))	6(6%)
Newborn weight, kg (mean ± SD)	3.4±0.4
Time to achieve the T4 level, s(median [interquartile range])	200.0 [187.0–326.0]
Loss of temperature sensation, segment (mean ± SD)	20.8±0.9
Loss of pinprick discrimination, segment (mean ± SD)	19.7±1.1

VCL: The distance from the middle of theC7 vertebra to the sacral hiatus; Segment: the total number of intrathecal bupivacaine block segments between the fifth sacral vertebra and the segment of cephalad spread.

Linear regression analysis showed that there was a significant univariate correlation between the spinal anesthesia spread and the parturient's height, weight, IAP, and VCL (all had *P*< 0.004, except for age, which had *P*> 0.05) (Table 2).

**Table 2:** Association between cephalad spread of spinal anesthesia and parturient characteristics.

	Loss of temperature sensation		Loss of pinprick discrimination	
	*r*	*P*	*r*	*P*
Age	0.149	0.122	0.154	0.110
Height	-0.437	<0.0001	-0.442	<0.0001
Weight	0.274	0.004	0.298	0.002
IAP	0.611	<0.0001	0.670	<0.0001
VCL	-0.681	<0.0001	-0.710	<0.0001

VCL: The distance from the middle of C7 vertebra to the sacral hiatus; *r*: correlation coefficient.

A multivariate analysis of predictors of successful anesthesia in all parturients showed that IAP and VCL were independently associated with the cephalad spread of spinal anesthesia (both *P* < 0.0001), whereas age, weight, and height could be excluded without changing the results (all *P*> 0.209) (Table 3).

**Table 3:** The combined linear regression of parturient characteristics against loss of temperature sensation and pinprick discrimination.

	Loss of temperature sensation				Loss of pinprick discrimination			
	*b*	*P*	95%CI		*b*	*P*	95%CI	
			Lower limit	Upper limit			Lower limit	Upper limit
Age	0.048	0.435	—-	—-	0.046	0.408	—-	—-
Height	-0.050	0.544	—-	—-	-0.034	0.642	—-	—-
Weight	0.095	0.215	—-	—-	0.086	0.209	—-	—-
IAP	0.226	<0.0001	0.154	0.298	0.326	<0.0001	0.244	0.408
VCL	-0.124	<0.0001	-0.156	-0.093	-0.160	<0.0001	-0.195	-0.125

VCL: The distance from the middle of theC7 vertebra to the sacral hiatus; *b*: regression coefficient;95%CI: 95% confidence interval of partial correlation coefficients.

There was a significant association between IAP and abdominal girth, with an estimated Spearman correlation coefficient of 0.534 (95% CI: 0.375–0.664), *P*<0.0001. The adjusted R^2^ was 0.688 for the regression equation.

Twenty-four parturients developed hypotension during surgery and were administered ephedrine (22.01%). Sinus bradycardia was found in 5 parturients (4.59%), and all were treated with atropine. Dyspnea was found in 6 parturients (5.50%), and who then received oxygen therapy.

## Discussion

In this prospective observational study, we included five parturient characteristics (age, weight, height, IAP, and VCL) to investigate the combined effect of parturient characteristics on the spread of spinal anesthesia. The most important finding was that IAP and VCL were both strongly correlated with a peak sensory block level in pregnant women who received single-shot spinal anesthesia for a cesarean section. The study suggests that parturients with increased IAP and shorter VCL often show a greater cephalad spread of spinal anesthesia.

Measuring IAP using bladder pressure is a well-established technique.[15] Chun et al.[14] established the method for measurement during pregnancy, emphasizing that the IAP in pregnant patients should be measured while they are in a supine position with a left lateral tilt of at least 10°. Thus, to avoid varying degrees of left lateral tilt, all parturients were measured while in a supine position with a left lateral tilt of 10°.

Our study found a strong positive correlation between IAP and the cephalad spread of spinal anesthesia in pregnant patients. Studies have shown that the extradural venous plexus is engorged when pregnant women lie in the supine position, due to inferior vena cava (IVC) obstruction by the enlarged uterus.[16–18] Compression causes the engorgement of veins in the lower part of the rigid vertebral canal, which leads to a displacement of lumbosacral CSF to the upper regions.[19] During pregnancy, the growing uterus, in addition to directly compressing the IVC, leads to a global increase in IAP, diverting blood into the vertebral venous system and leading to shrinkage of the dura on the subarachnoid space.[19]

Previous studies have speculated that CSF volume plays a major role in determining spinal spread and duration.[6, 7] Several factors may affect CSF volume in parturient. First, increased IAP has been demonstrated to increase IVC pressure in non-pregnant patients undergoing laparoscopy,[20] and thus, the increased IAP would cause the increase of IVC pressure, the same reaction can occur in pregnant patients. The increase of IVC pressure would cause the blood diverting into the vertebral venous system and displacing lumbosacral CSF. [16,19,21] Second, it has also been theorized that reduced CSF volume observed in pregnant patients may be due to increased intra-abdominal pressure caused by inward movement of soft tissue in the intervertebral foramen, which would displace lumbosacral CSF.[7, 22] However, Ozkan ST et al.,[23] found that IAP does not contribute to peak sensory block level in term parturients, which might be because they used hyperbaric local anesthetic. It is important to note that hyperbaric local anesthetic was reported to influence local anesthetic intrathecal spread.[24]

Logic might suggest that a longer VCL would lead to less cephalad spread of spinal anesthesia after administration of a fixed dose of bupivacaine. In our study, we found that VCL was another relevant primary factor of the spread of spinal anesthesia in term parturients. The results of the current study are similar to our previous study, which used non-pregnant patients.[11]

Our study showed that there was a significant association between IAP and abdominal girth. The study confirmed the hypothesis in our previous study that abdominal girth indirectly reflected the IAP.[11] Lee YH et al.[25] reported that abdominal girth was correlated with maximal spinal level in term parturients, a result also found in the present study. Thus, the abdominal girth (which was relatively easier to measure than IAP) could be used as a predictor of spinal anesthesia spread in pregnant patients. In the current study, BMI was not included in the multiple regression analysis, in which we used stepwise selection to test which factors were the primary predictors, because both the parturient's height and weight were included. Variables that were included in the multiple regression analysis must conform to the principle of independence, and BMI is calculated based on height and weight. Compared to BMI, IAP has a great predictive value for cephalad spread with plain bupivacaine in term parturients. This is likely because increased IAP can decrease lumbosacral CSF volume,[7] which is thought to be the main determinant of spinal anesthesia spread.[6] Previous studies found disparate results regarding the association between BMI and the intrathecal spread of injected local anesthetics, which can be explained by differing body shapes among individuals.[2,4,26] An increase in BMI may not always result in increased IAP.

There were some limitations of the present study. First, our study only showed that IAP has a positive correlation and VCL has a negative correlation with cephalad spread of spinal anesthesia, and the results did not suggest a specific dose of bupivacaine that would allow for achieving the anticipated block level, such as T4. The regression equation involving IAP, VCL, and a plain bupivacaine dose for T4 in term parturients needs further research. Second, in the current study, only plain bupivacaine was used, so in order to find out whether same findings are observed with hyperbaric bupivacaine, further study is needed. Third, the lack of IAP measurements before spinal anesthesia is also an important limitation. Fourth, further studies should be performed to measure the duration of spinal block, because adequate anesthesia for the duration of surgery is not just dependent on the initial block height achieved, but on the block height throughout.

## Conclusion

In conclusion, our data indicated that IAP and VCL have significant predictive values for cephalad spread of spinal anesthesia with plain bupivacaine, and there was a positive association between IAP and abdominal girth in term parturients. Parturients with greater IAP and shorter VCL may have greater cephalad spread after a dose of plain bupivacaine is administered.

## Supporting information

S1 File Protocol. (DOCX)

S2 File Research program. (DOC)

S3 File STROBE checklist. (DOCX)

S4 File Approved file of ethical committee. (PDF)
