# Supplementary material for: Intra-abdominal pressure, vertebral column length, and spread of spinal anesthesia in parturients undergoing cesarean section: An observational study
Source: PLoS One. 2018 Apr 3;13(4):e0195137. doi: 10.1371/journal.pone.0195137 (PMC5882131; doi:10.1371/journal.pone.0195137)
Supplement: S1 File — (DOCX) [file pone.0195137.s001.docx]

**Intra-abdominal Pressure, Vertebral Column Length, and Spread of Spinal Anesthesia** **for Cesarean Section**

**Abstract：**In parturients with increased intra-abdominal pressure (IAP) and short stature, a greater cephalad spread of spinal anesthesia after a fixed amount of plain bupivacaine is often observed. Thus, we designed this prospective study to test whether IAP and vertebral column length (VCL) were predictors of spinal spread in parturients for cesarean section.

**Background**

Regional anesthesia using local anesthetics is the preferred anesthetic technique for cesarean delivery. Plain bupivacaine is often used for spinal anesthesia. However, the spread of intrathecal plain bupivacaine is highly unpredictable. Many of the physiologic changes that occur during pregnancy increase the effect of a local anesthetic injection. Lumbosacral cerebrospinal fluid (CSF) volume and pressure were found to be the primary determinants that affect spinal cephalad spread, but that information had little practical value because these measurements were inconvenient to obtain. Patient characteristics such as height, weight, and body mass index are frequently used to predict spinal anesthesia spread, with unsatisfactory results.

A previous study found that abdominal girth and vertebral column length (VCL) have significant predictive value for the cephalad spread of spinal anesthesia in the term parturient. Increased abdominal girth has been reported to have a better correlation with increased intra-abdominal pressure (IAP) in non-pregnant patients, which might reduce lumbosacral CSF volume. However, abdominal girth reflects IAP indirectly. Factors such as pregnancy or morbid obesity may result in different IAPs among patients with the same abdominal girth. Thus, we aimed to investigate whether IAP and VCL were significant predictors of spinal spread of plain bupivacaine in term parturients.

**Purpose**

A previous study found that abdominal girth and vertebral column length (VCL) have significant predictive value for the cephalad spread of spinal anesthesia in the term parturient. However, abdominal girth reflects IAP indirectly. Thus, we aimed to investigate whether IAP and VCL were significant predictors of spinal spread of plain bupivacaine in term parturients.

**Materials and Methods**

**Study Type**

Observational

**Subjects**

**Inclusion Criteria**

- Age greater than 18 years old
- pregnant patients having single-shot spinal anesthesia for elective cesarean section

**Exclusion Criteria**

- Patients with pre-eclampsia
- diseases leading to peripheral edema or ascites
- contraindications for spine anesthesia,
- history of allergy to bupivacaine
- history of spinal puncture failure
- patients needed additional intra-operative analgesia.

**Procedure**

All patients fasted for 8-10h before surgery. After the patient entered the operating room, intravenous access was established, Ringer’s lactate 500mL was preloaded, and standard monitoring was started. The patient was placed supine on a horizontal operating table, and the abdominal girth was measured at the level of umbilicus during the end of expiration. VCL was measured from the C7 vertebra to the sacral hiatus. After the L3-L4 interspace was confirmed, a 25-gauge Quincke needle was inserted using a midline approach with the bevel cephalad. When free flow of CSF was obtained, 2mL of 0.5% plain bupivacaine was injected into the subarachnoid space over 10 seconds (C7-SH).Patients were then positioned in a 10° Trendelenburg position with 10° left-lateral tilt. The cephalad spread of spinal anesthesia was assessed in both midclavicular lines using ice for loss of temperature sensation and an 18-gauge needle for loss of pinprick sensation every 1minute.When a bilateral sensory block of cold sensation to the T4 dermatome level was achieved, surgery commenced, and the operating table was returned to a horizontal position while maintaining left lateral tilt. Further, after establishing a T4 sensory level, a transurethral catheter was inserted to drain the bladder and measure IAP in the supine position with 10° left lateral tilt as described by Chun et al.

Sensory block assessment continued every 2 minutes until the block level remained unchanged for three consecutive assessments. The total number of spinal anesthesia block segments was recorded from the fifth sacral vertebra to the segment of spinal anesthesia spread. General anesthesia was induced if spinal administration failed to produce surgical anesthesia.

All anesthesia procedures were performed by the same attending physician, and the assessment of cephalad spread of spinal anesthesia was completed by another anesthetist who was blinded to the parturient’s measurement information. Hypotension was defined as a decrease in systemic arterial pressure (SAP) of greater than 30% below baseline or to less than 90 mmHg and was treated with 5mg of ephedrine intravenously. Bradycardia was defined as heart rate less than 50 beats/min and was treated with intravenous atropine

**Measurements**

Parturient demographic variables, including age, height, weight, length of gestation, gravidity, parity, number of fetuses, IAP, and VCL were recorded. We also recorded maximum sensory block level, including loss of temperature sensation and pinprick discrimination level, and neonatal weight.

**Statistical Analysis**

**Sample Size Calculation**

Five spinal anesthesia spread predictors were used in this study. If the anticipated effect size was 0.15, the desired statistical power level was 0.8, the predictors were 5, and the minimum required sample size was 92, with a probability level of 0.05.

date

**Date Analysis**

Statistical analysis was performed with SPSS software (version 19.0, SPSS Inc., Chicago, IL, USA). A linear regression analysis was used to determine the correlation of the spinal anesthesia-induced loss of temperature sensation and pinprick discrimination with age, weight, height, body mass index, IAP, and VCL. During multiple regression analysis, stepwise selection was performed to test which factors were the primary predictors. R2 is the multiple correlation coefficient of determination. A P value < 0.05 was considered to be statistically significant.

[**Technological**](javascript:void(0);) **Route**

Spinal Anesthesia

 puncture at L3-L4 interspace, inject 2mL of 0.5% plain bupivacaine into the subarachnoid space

Exclusion Criteria：

pre-eclampsia

peripheral edema or ascites

contraindications for spine anesthesia

history of allergy to bupivacaine

patients need additional intra- operative analgesia

assess the cephalad spread of spinal anesthesia，and record five parturient demographic variables

dates are analyzed by linear regression analysis and multiple regression

Exclude：

Spinal puncture failure

The subarachnoid block is invalid

**References**

[1] Ozkan ST, Orhan-Sungur M, Basaran B, et al. The effect of intra-abdominal pressure on sensory block level of single-shot spinal anesthesia for cesarean section: an observational study. Int J Obstet Anesth. 2015. 24(1): 35-40.

[2] Chun R, Baghirzada L, Tiruta C, Kirkpatrick AW. Measurement of intra-abdominal pressure in term pregnancy: a pilot study. Int J Obstet Anesth. 2012. 21(2): 135-9.

[3] Zhou QH, Zhu B, Wei CN, Yan M. Abdominal girth and vertebral column length can adjust spinal anesthesia for lower limb surgery, a prospective, observational study. BMC Anesthesiol. 2016. 16: 22.

[4] Ngaka TC, Coetzee JF, Dyer RA. The Influence of Body Mass Index on Sensorimotor Block and Vasopressor Requirement During Spinal Anesthesia for Elective Cesarean Delivery. Anesth Analg. 2016. 123(6): 1527-1534.

[5] 熊威威, 蒋奕红, 庾俊雄, 于俊芳, 赵振海. 影响剖宫产术中腰麻最高痛觉消失平面的相关因素研究. 临床合理用药杂志. 2013. 6(6): 16-17.

[6] Kirkpatrick AW, Roberts DJ, De Waele J, et al. Intra-abdominal hypertension and the abdominal compartment syndrome: updated consensus definitions and clinical practice guidelines from the World Society of the Abdominal Compartment Syndrome. Intensive Care Med. 2013. 39(7): 1190-206.

[7] Pitkänen MT. Body mass and spread of spinal anesthesia with bupivacaine. AnesthAnalg.

1987;66(2): 7-131.

[8] Ngaka TC, Coetzee JF, Dyer RA. The Influence of Body Mass Index on Sensorimotor Block

and Vasopressor Requirement During Spinal Anesthesia for Elective Cesarean Delivery.

AnesthAnalg. 2016;123(6): 1527-1534.

[9] Wei CN, Zhang YF, Xia F, Wang LZ, Zhou QH. Abdominal girth, vertebral column length and

spread of intrathecal hyperbaric bupivacaine in the term parturient. Int J ObstetAnesth.

2017; Feb 27.pii: S0959-289X(16)30184-4.

[10] Kirkpatrick AW, Roberts DJ, De Waele J, et al. Intra-abdominal hypertension and the

abdominal compartment syndrome: updated consensus definitions and clinical practice

guidelines from the World Society of the Abdominal Compartment Syndrome.

Intensive Care Med. 2013;39(7): 1190-1206.
