# Supplementary material for: Intra-abdominal pressure, vertebral column length, and spread of spinal anesthesia in parturients undergoing cesarean section: An observational study
Source: PLoS One. 2018 Apr 3;13(4):e0195137. doi: 10.1371/journal.pone.0195137 (PMC5882131; doi:10.1371/journal.pone.0195137)
Supplement: S2 File — (DOC) [file pone.0195137.s002.doc]

**研究计划书**

**剖宫产产妇腹内压和脊柱长度对蛛网膜下腔阻滞麻醉后感觉阻滞平面的影响**

**研究方案**

1. **研究背景**

蛛网膜下腔阻滞麻醉因起效快，效果确切，广泛应用于剖宫产手术。产妇由于生理、激素、解剖学的改变，剖宫产时对单次蛛网膜下腔阻滞麻醉局麻药的需求减少，同时蛛网膜下腔局麻药的扩散和感觉阻滞平面也更加难以预测。

研究表明，产妇增大的子宫压迫下腔静脉导致硬膜外静脉丛扩张和硬膜外血容量增加，使得腰骶部脑脊液容量下降，从而增加了蛛网膜下腔局麻药的扩散，减少局麻药的需求。增大的子宫同时可引起腹围和腹内压的明显增加，可能是增加蛛网膜下腔局麻药扩散的另一个机制。一方面，腹内压增加间接导致下腔静脉压力增加和硬膜外静脉丛扩张，腰骶部脑脊液容量减少；另一方面，腹内压增加使得腹膜后软组织向椎间孔方向移动，压迫硬脊膜。

逻辑上，身高较高的患者蛛网膜下腔给与一定剂量的局部麻醉药更少的往头部扩散。但很多研究也表明身高对蛛网膜下腔局部麻醉药扩散影响微小。这是由于大部分成年人之间的身高差是由于下肢长骨的长度而不是脊柱。较多研究表明，患者的脊柱长度与蛛网膜下腔局部麻醉药扩散具有一定相关性。

故本研究利用多元线性回归分析，探讨剖宫产产妇年龄、身高、体重、腹内压和脊柱长度等体格特征中，腹内压和脊柱长度能否对蛛网膜下腔0.5%等比重布比卡因阻滞后感觉阻滞平面产生明显影响。

1. **研究目的**

探讨剖宫产产妇年龄、身高、体重、腹内压和脊柱长度等体格特征中，腹内压和脊柱长度能否对蛛网膜下腔0.5%等比重布比卡因阻滞后感觉阻滞平面产生明显影响。

1. **研究设计和方法：**

（1）前瞻性观察研究，单中心实验。

（2）入选标准：① 择期接受蛛网膜下腔阻滞麻醉剖宫产的产妇；② 年龄＞18岁

（3）排除标准： ① 合并子痫前期的患者；② 疾病引起外周水肿和腹水的患者；③ 蛛网膜下腔阻滞麻醉禁忌症； ④ 布比卡因过敏患者；⑤ 不能配合完成麻醉平面测试的患者；

（4）操作流程：患者进入手术室前常规禁食8-10小时，禁饮4-6小时。进入手术室后 连续监测心电图、无创血压、呼吸频率和指脉搏血氧饱和度，开放上肢静脉通路，预输注500ml乳酸林格氏液。患者平卧位，测腹围，宫高。左侧卧位，正中法，25G Quincke型蛛网膜下腔阻滞穿刺针， L3/4椎间隙行蛛网膜下腔阻滞， 0.5%等比重布比卡因2mL，局麻药温度接近体温，20s输注完毕。操作完，产妇平卧，摇床左倾15°。患者阻滞平面的测量采用温度觉和痛觉消失法，每1min测定1次阻滞平面。当温度觉阻滞平面到达T4时，记录蛛网膜下腔局麻药注射完毕到温度觉消失平面达到T4时间（T4平面时间）。并改为每2分钟测量1次平面，直到连续3次测量阻滞平面没有明显改变，记录最高阻滞平面，包括温度觉消失平面和痛觉消失平面。记录切皮前测定运动阻滞程度和手术结束时运动阻滞程度。

患者温度觉阻滞平面达到T4时, 经尿道膀胱插入Forley尿管，排空膀胱后，将25 mL无菌等渗盐水经尿管注入膀胱内，夹住尿管，连接尿管与尿袋，在尿管与引流袋之间连接三通接头，接压力计进行测定，以腋中线为调零点，测定切皮前腹内压。手术结束时再次测定腹内压。

如果发生低血压（收缩压<90mmHg或较基础血压下降>30%）,经静脉注射麻黄碱5mg，并加快补液；如果发生心动过缓（<50 次/min），静脉注射阿托品0.5mg。

（5）观察指标：记录患者年龄、身高、体重、脊柱长度、宫高、腹围、入手术室收缩压、舒张压和平均压、孕次、产次、孕周、胎儿数、新生儿体重和新生儿1min、5min Apgar评分;记录切皮前腹内压和手术结束时腹内压、T4平面时间、最高阻滞平面包括温度觉消失平面和痛觉消失平面、切皮前和手术后运动阻滞程度；记录手术时间、出血量、液体输入量、不良反应包括恶心、呕吐、寒战；记录麻黄碱和阿托品使用情况、改为全身麻醉患者例数。

（6）实验操作流程图

蛛网膜下腔阻滞麻醉

剖宫产手术患者

在L3/4椎间隙穿刺，蛛网膜下腔注入0.5%等比重布比卡因2mL

排除：

合并严重心血管疾病患者

疾病引起外周水肿和腹水的患者

明确的椎管狭窄病史

布比卡因过敏患者

蛛网膜下腔阻滞麻醉禁忌症

不能配合完成麻醉平面测试的患者

在两侧锁骨中线测量温度觉和痛觉消失平面，记录患者五个身体特征指标

采用双变量线性和多元线性回归分析进行统计处理

剔除：

L3/4椎间隙穿刺失败

蛛网膜下腔阻滞无效

双侧阻滞平面不均衡

得出与剖宫产产妇蛛网膜下腔阻滞后感觉阻滞平面明显相关的指标

**3，样本量计算和统计学处理**

样本量计算采用G-Power3.1.9.2 计算，本研究纳入了患者年龄、身高、体重、腹内压和脊柱长度五个身体特征预测指标。多元线性回归分析时，通常情况下预期效应值为0.15即认为是中等的和合适的，设置检验水准为0.05，检验效能为0.8，故最少样本量为92。

采用spss18.0统计软件进行数据分析。主要的统计学研究采用双变量线性相关对温度觉和痛觉消失平面与年龄、身高、体重、腹内压和脊柱长度五个预测指标分别进行相关性分析。应用多元线性回归分析对年龄、身高、体重、腹内压和脊柱长度五个预测指标与温度觉和痛觉消失平面进行回归分析，分析这五个因素对单次蛛网膜下腔阻滞麻醉后感觉阻滞平面的影响大小，多元线性回归分析时采用逐步法，排除与局部麻醉药扩散关系不明显的因素。r为双变量相关时的相关系数，R^2^多元线性回归方程的决定系数。

**参考文献**

[1] Ozkan ST, Orhan-Sungur M, Basaran B, et al. The effect of intra-abdominal pressure on sensory block level of single-shot spinal anesthesia for cesarean section: an observational study. Int J Obstet Anesth. 2015. 24(1): 35-40.

[2] Chun R, Baghirzada L, Tiruta C, Kirkpatrick AW. Measurement of intra-abdominal pressure in term pregnancy: a pilot study. Int J Obstet Anesth. 2012. 21(2): 135-9.

[3] Zhou QH, Zhu B, Wei CN, Yan M. Abdominal girth and vertebral column length can adjust spinal anesthesia for lower limb surgery, a prospective, observational study. BMC Anesthesiol. 2016. 16: 22.

[4] Ngaka TC, Coetzee JF, Dyer RA. The Influence of Body Mass Index on Sensorimotor Block and Vasopressor Requirement During Spinal Anesthesia for Elective Cesarean Delivery. Anesth Analg. 2016. 123(6): 1527-1534.

[5] 熊威威, 蒋奕红, 庾俊雄, 于俊芳, 赵振海. 影响剖宫产术中腰麻最高痛觉消失平面的相关因素研究. 临床合理用药杂志. 2013. 6(6): 16-17.

[6] Kirkpatrick AW, Roberts DJ, De Waele J, et al. Intra-abdominal hypertension and the abdominal compartment syndrome: updated consensus definitions and clinical practice guidelines from the World Society of the Abdominal Compartment Syndrome. Intensive Care Med. 2013. 39(7): 1190-206.

[7] Pitkänen MT. Body mass and spread of spinal anesthesia with bupivacaine. AnesthAnalg. 1987;66(2): 127-131.

[8] Ngaka TC, Coetzee JF, Dyer RA. The Influence of Body Mass Index on Sensorimotor Block and Vasopressor Requirement

During Spinal Anesthesia for Elective Cesarean Delivery. AnesthAnalg. 2016;123(6): 1527-1534.

[9] Wei CN, Zhang YF, Xia F, Wang LZ, Zhou QH. Abdominal girth, vertebral column length and spread of intrathecal hyperbaric

bupivacaine in the term parturient. Int J ObstetAnesth. 2017; Feb 27.pii: S0959-289X(16)30184-4.

[10] Kirkpatrick AW, Roberts DJ, De Waele J, et al. Intra-abdominal hypertension and the abdominal compartment syndrome: updated

consensus definitions and clinical practice guidelines from the World Society of the Abdominal Compartment Syndrome. Intensive

Care Med. 2013;39(7): 1190-1206.
