# Supplementary material for: Intra-abdominal pressure, vertebral column length, and spread of spinal anesthesia in parturients undergoing cesarean section: An observational study
Source: PLoS One. 2018 Apr 3;13(4):e0195137. doi: 10.1371/journal.pone.0195137 (PMC5882131; doi:10.1371/journal.pone.0195137)
Supplement: S4 File — (PDF) [file pone.0195137.s004.pdf]

（2017年3月10日）

临床科研伦理审查送审文件清单及接收表

项目名称：　　　　　　　　　　　　　　　　　　　　编号：2017０４０1

序号	文件	核对
1	送审项目资料清单	
2	宁波市第七医院人体研究申请表（申请者签名、专业组负责人或科主任签名，并注明日期）	
3	临床试验方案（注明版本号/日期，研究者签名）	
4	受试者知情同意书	
5	病例报告表等其他相关资料	
6	多中心试验需提供其他参与单位列表及中心伦理批件	
7	主要研究人员	
8	主要研究者简历及参加研究人员简介	
9	临床研究项目负责人承诺书、宁波市第七医院临床研究者岗位职责	
10	临床研究申办者承诺书及承担研究相关损害赔偿的说明，如有保险，附保险证明	
11	其它资料	

宁波市第七医院医院人体研究伦理委员会声明：对提交的有关伦理审查的申请材料（包括电子版），本委员会只用于伦理审查，不以任何其它目的使用相关材料。一旦伦理审核结束，除留存一份完整的材料作为备案以外，剩余资料均归还申办方或研究者。未经授权，本伦理委员会及其成员均严格遵守保密约定，承当由于泄密导致的一切法律责任。

宁波市第七医院人体研究伦理委员会

接收人签名：[illegible]　　　　日期：2017.4.24

（2017年3月10日）

宁波市第七医院 人体研究申请表

编号：20170401

<table>
<tr><td rowspan="5">项目情况</td><td colspan="5">项目名称：剖宫产产妇腹内压和脊柱长度对蛛网膜下腔阻滞麻醉后感觉阻滞平面的影响</td></tr>
<tr><td colspan="5">申请类型 ☐ 药/械临床试验 ☑ 临床科研 ☐ 医疗新技术 ☐ 上市后产品研究</td></tr>
<tr><td>药/械类别</td><td>/</td><td>规格：/</td><td>试验类别</td><td>前瞻性观察性临床试验</td></tr>
<tr><td colspan="5">适应症：剖宫产手术的患者</td></tr>
<tr><td colspan="5">本院承担例数： 研究总经费：自筹</td></tr>
<tr><td rowspan="2">申办方</td><td colspan="5">申办单位：宁波市第七医院麻醉科</td></tr>
<tr><td colspan="5">联系人：倪婷婷 联系电话：13732130748</td></tr>
<tr><td>CRO</td><td colspan="5">名称：无
联系人： / 联系电话：/</td></tr>
<tr><td rowspan="3">研究单位</td><td colspan="5">研究单位（科室）：宁波市第七医院麻醉科 负责单位：是☑ 否☐</td></tr>
<tr><td colspan="5">专业组负责人或科主任统筹安排项目负责人及参加研究人员（事先识别参加研究人员的利益冲突，按具体分工和职责排序）：

参加项目研究人员：倪婷婷 潘应锋 贺波 雍安翠 王璐 田鑫

项目负责人签名：倪婷婷 日期：2017.4.10</td></tr>
<tr><td colspan="5">专业组负责人或科主任签名：[illegible] 日期：2017年4月12日</td></tr>
<tr><td colspan="6">相关职能部门审批意见：（药/械临床试验由临床药理审批；临床科研和上市后研究由科教部审批；医疗新技术由医务部审批）
[illegible]
签名：[illegible] 日期：2017年[illegible]月[illegible]日</td></tr>
<tr><td colspan="6">伦理委员会意见：
签名：[illegible]、[illegible] 日期：2017年5月9日
（印章：宁波市第七医院医学伦理委员会）</td></tr>
<tr><td colspan="6">主管院领导审批意见
签名：[illegible] 日期：2017年5月9日</td></tr>
</table>

研究方案

1， 研究背景

蛛网膜下腔阻滞麻醉因起效快，效果确切，广泛应用于剖宫产手术。产妇由于生理、激素、解剖学的改变，剖宫产时对单次蛛网膜下腔阻滞麻醉局麻药的需求减少，同时蛛网膜下腔局麻药的扩散和感觉阻滞平面也更加难以预测。

研究表明，产妇增大的子宫压迫下腔静脉导致硬膜外静脉丛扩张和硬膜外血容量增加，使得腰骶部脑脊液容量下降，从而增加了蛛网膜下腔局麻药的扩散，减少局麻药的需求。增大的子宫同时可引起腹围和腹内压的明显增加，可能是增加蛛网膜下腔局麻药扩散的另一个机制。一方面，腹内压增加间接导致下腔静脉压力增加和硬膜外静脉丛扩张，腰骶部脑脊液容量减少；另一方面，腹内压增加使得腹膜后软组织向椎间孔方向移动，压迫硬脊膜。

逻辑上，身高较高的患者蛛网膜下腔给与一定剂量的局部麻醉药更少的往头部扩散。但很多研究也表明身高对蛛网膜下腔局部麻醉药扩散影响微小。这是由于大部分成年人之间的身高差是由于下肢长骨的长度而不是脊柱。较多研究表明，患者的脊柱长度与蛛网膜下腔局部麻醉药扩散具有一定相关性。

故本研究利用多元线性回归分析，探讨剖宫产产妇年龄、身高、体重、腹内压和脊柱长度等体格特征中，腹内压和脊柱长度能否对蛛网膜下腔 0.5%等比重布比卡因阻滞后感觉阻滞平面产生明显影响。

2， 研究目的

利用多元线性回归分析，探讨剖宫产产妇年龄、身高、体重、腹内压和脊柱长度等体格特征中，腹内压和脊柱长度能否对蛛网膜下腔 0.5%等比重布比卡因阻滞后感觉阻滞平面产生明显影响。

3， 研究设计和方法：

（1）前瞻性观察研究，单中心实验。

（2）入选标准：① 择期接受蛛网膜下腔阻滞麻醉剖宫产的产妇；② 年龄＞20 岁

（3）排除标准：① 合并子痫前期的患者；② 疾病引起外周水肿和腹水的患者；③ 蛛网膜下腔阻滞麻醉禁忌症；④ 布比卡因过敏患者；⑤ 不能配合完成麻醉平面测试的患者；

（4）操作流程：患者进入手术室前常规禁食 8-10 小时，禁饮 4-6 小时。进入手术室后连续监测心电图、无创血压、呼吸频率和指脉搏血氧饱和度，开放上肢静脉通路，预输注 500ml 乳酸林格氏液。患者平卧位，测腹围，宫高。左侧卧位，正中法，25G Quincke 型蛛

网膜下腔阻滞穿刺针， L3/4 椎间隙行蛛网膜下腔阻滞， 0.5%等比重布比卡因 11mg，局麻药温度接近体温，20s 输注完毕。操作完，产妇平卧，摇床左倾 15°。患者阻滞平面的测量采用温度觉和痛觉消失法，每 30 秒测定 1 次阻滞平面。当温度觉阻滞平面到达 T_4时，记录蛛网膜下腔局麻药注射完毕到温度觉消失平面达到 T_4时间（T_4平面时间）。并改为每 2 分钟测量 1 次平面，直到连续 3 次测量阻滞平面没有明显改变，记录最高阻滞平面，包括温度觉消失平面和痛觉消失平面。记录切皮前测定运动阻滞程度和手术结束时运动阻滞程度。

患者温度觉阻滞平面达到T_4时，经尿道膀胱插入Forley尿管，排空膀胱后，将25 mL无菌等渗盐水经尿管注入膀胱内，夹住尿管，连接尿管与尿袋，在尿管与引流袋之间连接三通接头，接压力计进行测定，以腋中线为调零点，测定切皮前腹内压。手术结束时再次测定腹内压。

如果发生低血压（收缩压<90mmHg 或较基础血压下降>30%），经静脉注射麻黄碱 5mg，并加快补液；如果发生心动过缓（<50 次/min），静脉注射阿托品 0.5mg。

（5）观察指标：记录患者年龄、身高、体重、脊柱长度、宫高、腹围、入手术室收缩压、舒张压和平均压、孕次、产次、孕周、胎儿数、新生儿体重和新生儿 1min、5min Apgar 评分;记录切皮前腹内压和手术结束时腹内压、T4 平面时间、最高阻滞平面包括温度觉消失平面和痛觉消失平面、切皮前和手术后运动阻滞程度；记录手术时间、出血量、液体输入量、不良反应包括恶心、呕吐、寒战；记录麻黄碱和阿托品使用情况、改为全身麻醉患者例数。

（6）实验操作流程图

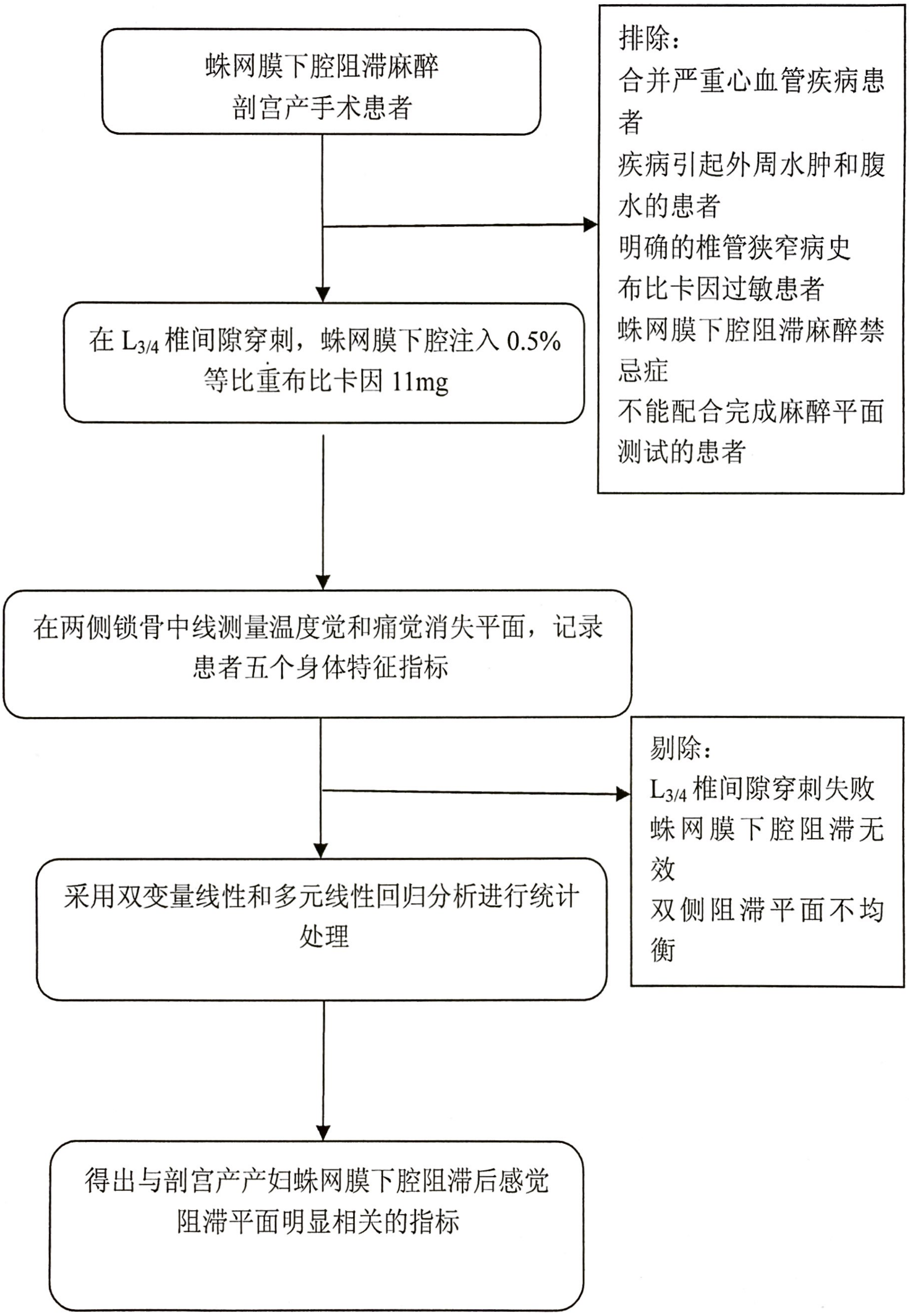
蛛网膜下腔阻滞麻醉
剖宫产手术患者
排除：
合并严重心血管疾病患者
疾病引起外周水肿和腹水的患者
明确的椎管狭窄病史
布比卡因过敏患者
蛛网膜下腔阻滞麻醉禁忌症
不能配合完成麻醉平面测试的患者
在 $L_{3/4}$ 椎间隙穿刺，蛛网膜下腔注入 0.5% 等比重布比卡因 11mg
在两侧锁骨中线测量温度觉和痛觉消失平面，记录患者五个身体特征指标
剔除：
$L_{3/4}$ 椎间隙穿刺失败
蛛网膜下腔阻滞无效
双侧阻滞平面不均衡
采用双变量线性和多元线性回归分析进行统计处理
得出与剖宫产产妇蛛网膜下腔阻滞后感觉阻滞平面明显相关的指标

3，样本量计算

样本量计算采用 G-Power3.1.9.2 计算，本研究纳入了患者年龄、身高、体重、腹内压和脊柱长度五个身体特征预测指标。多元线性回归分析时，通常情况下预期效应值为 0.15 即认为是中等的和合适的，设置检验水准为 0.05，检验效能为 0.8，故最少样本量为 92。

4，数据管理和保密

每例受试者 1 份病例报告表，由研究人员填写并由专人统一保存，并录入 Excel 表格，专人管理。有关受试者身份的所有记录均予以保密，并且在相关法律和/或法规允许的范围之内这些资料不对外公开。

匿名方式收集数据，给数据处理中心的病倒报告和任何试验文书不会出现患者姓名，所有数据将被保密。研究负责人保证所有临床实验材料和数据将根据法律按照匿名进行科学处理。

实验室数据将保存在一个单独、安全的地方,只有相关的实验室人员能进入。任何公布的数据将遵守数据保护相关法律和/或法规，包括受试者和调查人员。

5，知情同意

受试者实施研究步骤前必须获得书面的知情同意，告知患者研究相关的具体内容，详见知情同意书。

术前调查者告诉患者试验性质，目的，可能的益处和风险。每个患者必须签字同意才能参加试验。签字前给患者足够的时间来决定是否参加和澄清任何问题。匿名方式收集数据，给数据处理中心的病倒报告和任何试验文书不会出现患者姓名，所有数据将被保密。

病人和调查者一起签署知情同意书。原件交由调查者，患者保留复印件。

6，不良事件呈报

蛛网膜下腔阻滞麻醉为剖宫产时常见麻醉方法，监测腹内压方法以往研究未见不良反应。任何研究调查者任何时间发现怀疑有不良事件或对继续实验有任何伦理问题是需要立即通知主持者。负责单位根据与主持者、质量和安全委员会或统计学家协商决定是否提前终止临床试验。

宁波市第七医院人体研究知情同意书

剖宫产产妇腹内压和脊柱长度对蛛网膜下腔阻滞麻醉后感觉阻滞平面的影响

尊敬的患者：

我们邀请您参加一项“剖宫产产妇腹内压和脊柱长度对蛛网膜下腔阻滞麻醉后感觉阻滞平面的影响”的临床研究，在您决定是否参加这项研究之前，请仔细阅读以下内容，它可以帮助您了解该项研究以及为什么要进行这项研究，研究的程序和期限，参加研究后可能给您带来的益处、风险和不便。

以下是本项研究的介绍：

一、研究背景和研究目的

研究背景：

蛛网膜下腔阻滞麻醉因起效快，效果确切，广泛应用于剖宫产手术。产妇由于生理、激素、解剖学的改变，剖宫产时对单次蛛网膜下腔阻滞麻醉局麻药的需求减少，同时蛛网膜下腔局麻药的扩散和感觉阻滞平面也更加难以预测。

研究表明，产妇增大的子宫压迫下腔静脉导致硬膜外静脉丛扩张和硬膜外血容量增加，使得腰骶部脑脊液容量下降，从而增加了蛛网膜下腔局麻药的扩散，减少局麻药的需求。增大的子宫同时可引起腹围和腹内压的明显增加，可能是增加蛛网膜下腔局麻药扩散的另一个机制。一方面，腹内压增加间接导致下腔静脉压力增加和硬膜外静脉丛扩张，腰骶部脑脊液容量减少；另一方面，腹内压增加使得腹膜后软组织向椎间孔方向移动，压迫硬脊膜。

逻辑上，身高较高的患者蛛网膜下腔给与一定剂量的局部麻醉药更少的往头部扩散。但很多研究也表明身高对蛛网膜下腔局部麻醉药扩散影响微小。这是由于大部分成年人之间的身高差是由于下肢长骨的长度而不是脊柱。较多研究表明，患者的脊柱长度与蛛网膜下腔局部麻醉药扩散具有一定相关性。

故本研究利用多元线性回归分析，探讨剖宫产产妇年龄、身高、体重、腹内压、脊柱长度等体格特征中，腹内压和脊柱长度能否对蛛网膜下腔 0.5%等比重布比卡因阻滞后感觉阻滞平面产生明显影响。

研究目的：

探讨剖宫产产妇年龄、身高、体重、腹内压和脊柱长度等体格特征中，腹内压和脊柱长度能否对蛛网膜下腔 0.5%等比重布比卡因阻滞后感觉阻滞平面产生

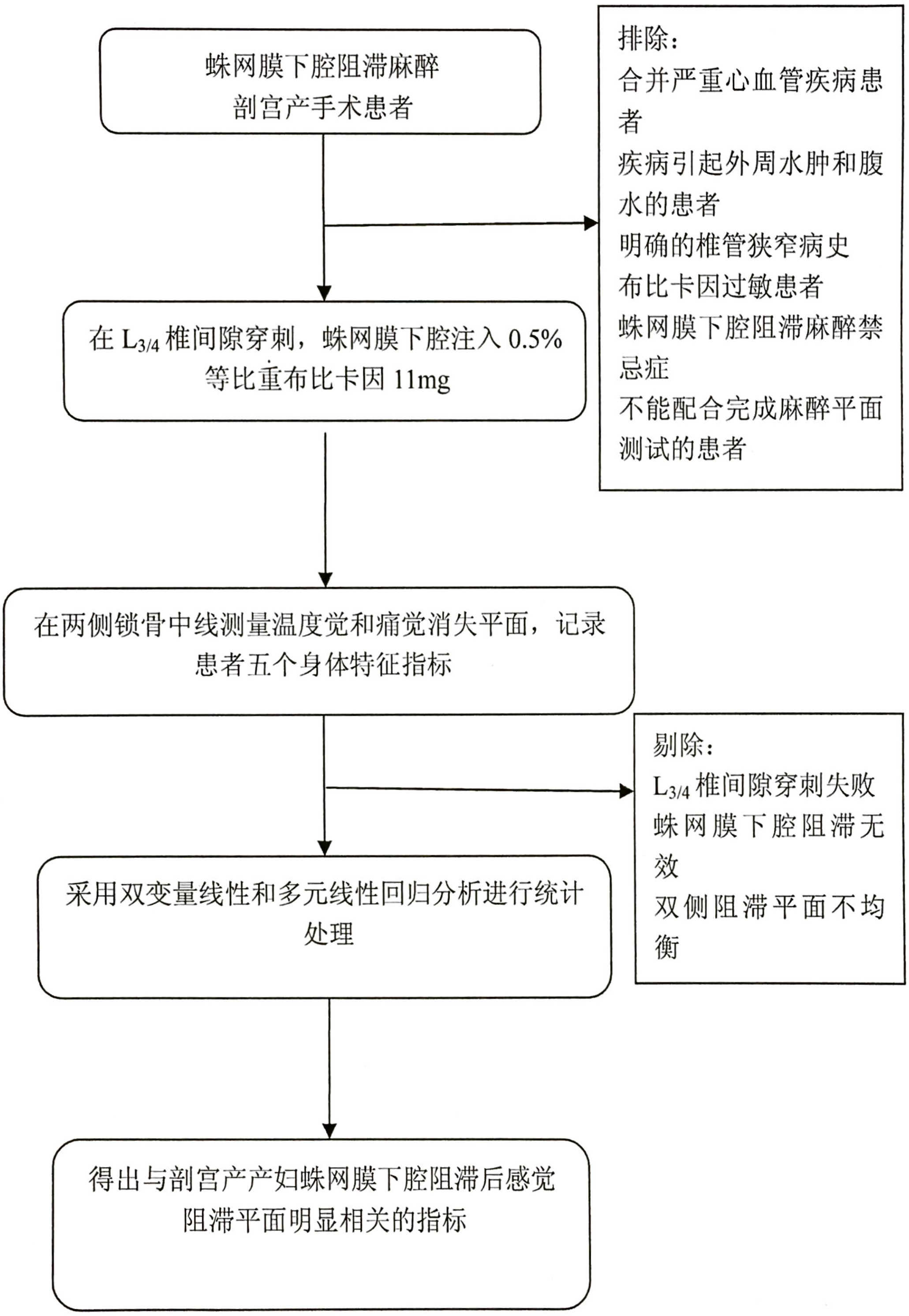

蛛网膜下腔阻滞麻醉
剖宫产手术患者
排除：
合并严重心血管疾病患者
疾病引起外周水肿和腹水的患者
明确的椎管狭窄病史
布比卡因过敏患者
蛛网膜下腔阻滞麻醉禁忌症
不能配合完成麻醉平面测试的患者
在 $L_{3/4}$ 椎间隙穿刺，蛛网膜下腔注入 0.5% 等比重布比卡因 11mg
在两侧锁骨中线测量温度觉和痛觉消失平面，记录患者五个身体特征指标
剔除：
$L_{3/4}$ 椎间隙穿刺失败
蛛网膜下腔阻滞无效
双侧阻滞平面不均衡
采用双变量线性和多元线性回归分析进行统计处理
得出与剖宫产产妇蛛网膜下腔阻滞后感觉阻滞平面明显相关的指标

研究起讫时间：从手术日至出院日。

随访时间：随访至出院时。

三、如果参加研究您需要做什么

1）按照临床常规要求，配合医护人员完成相关的术前准备；

2）如实提供自身疾病和相关检查治疗情况，以利于研究人员准确地进行研究相关的评估；

3) 如果您符合研究纳入标准，您可自愿参加研究，并签署知情同意书。

4）因在研究过程中我们将观察记录您的麻醉阻滞平面、体格特征指标及不良反应发生情况，请配合医护人员对您的随访询问。

四、参加本研究可能给您带来的受益

1. 个人受益：合适的麻醉平面能使剖宫产手术顺利进行，同时可以减少术后并发症及不良反应的发生，促进术后恢复。

2. 社会受益：本研究立足于剖宫产产妇蛛网膜下腔阻滞扩散平面难以准确预测，试图分析年龄、身高、体重、腹内压和脊柱长度五个预测指标与温度觉和痛觉消失平面的影响大小，为临床麻醉提供依据。

五、参加本研究可能发生的不良反应、风险以及风险防范措施

蛛网膜下腔阻滞麻醉为剖宫产时常见麻醉方法，监测腹内压方法以往研究未见不良反应。任何研究调查者任何时间发现怀疑有不良事件或对继续实验有任何伦理问题会立即通知主持者（倪婷婷、13732130748）。

如蛛网膜下腔阻滞不能满足术中要求及手术顺利进行，可硬膜外推注补救剂量或者全身麻醉下进行子宫下段剖宫产术，不会影响手术顺利进行及术后镇痛。

六、费用情况说明

参与本研究较临床常规未增加药物费用，本研究不需要额外的预算。受试者和医疗保险部门或第三方支付者将不会为研究相关医疗付费。研究机构支付研究相关支持（包括中心实验室）。受试者和医疗保险部门或第三方支付者需要为常规医疗或与研究无关的医疗进行付费。这些常规医疗包括住院医疗和出院时带药或其它医疗。

七、参与研究的补偿，包括损伤的赔偿

本研究属于观察性研究，较临床常规未增加药物费用和增加临床治疗风险，自愿参加研究，无相关经济补偿。

八、替代方案

若您不愿意参加本研究，不会影响您的常规临床治疗，不存在替代方案。

九、您个人信息的保密

您的医疗记录（包括研究病历及理化检查报告等）将按规定保存在医院。除研究者、伦理委员会、监查、稽查、药政管理部门等相关人员将被允许查阅您的医疗记录外，其他与研究无关的人员在未得到允许的情况下，无权查阅您的医疗记录。本研究结果的公开报告将不会披露您的个人身份。我们将在允许的范围内，尽一切努力保护您个人医疗资料的隐私。

十、终止参加研究

是否参加本项研究完全取决于您的自愿。您可以拒绝参加此项研究，或在研究过程中的任何时间无理由退出研究，这都不会影响您和医生的关系，都不会影响对您的医疗或有其他方面利益的损失。此外，由于以下原因，可能会终止您参与本研究：

1、您未遵从研究人员及临床治疗医生的相关要求。

2、您的疾病发生了变化，可能需要接受别的治疗。

十一、伦理委员会

本研究已向宁波市第七医院人体研究伦理委员会报告，经委员会的全面审查和包括对受试者的风险评估，并获得了批准。在研究中过程中，**有关伦理和权益事宜可联系宁波市第七医院人体研究伦理委员会，电话：白天:0574-86655001；晚上（总值班）：0574-86655001；邮箱地址： dqyykjk@163.com**

我确认已阅读并理解了本研究的知情同意书，自愿接受本研究中的治疗方法，并同意将我的医疗数据用于本研究的发表。

受试者签名：__________ 联系方式：______________ 日期：___

代理人签名：______________与受试者关系______________联系方式

日期______________

（如果需要）

见证人（如果需要）：____________联系方式：_____________ 日期：___

我确认已向患者解释了本研究的详细情况，包括其权利以及可能的受益和风险，并给其一份签署过的知情同意书副本。

研究者签名：____________

联系方式：____________（手机）　　　　日期：________

病例报告表

Checklist

请在最后完成整本调查表时再根据 Checklist 仔细核对是否填写完全，在填写完整的各部分前打钩

□术前访视：已详细向患者告知本研究详细内容，并获得患者的知情同意及签字确认。

□围手术期资料

□随访资料

□已完成上述各项登记填写，完成日期：____年____月____日

监察者：________

信息首页

剖宫产产妇腹内压和脊柱长度对蛛网膜下腔阻滞麻醉后感觉阻滞平面的影响—前瞻观察性、单中心研究

（病例登记表）

病例姓名：____________________

签署知情同意书日期：__________

Part 1

手术及围手术期资料

（登记医生填写部分）

登记者：____________

日期：____________

入选标准及排除标准

	入选标准	
	是	否
年龄：≥20岁		
择期行剖宫产手术		
患者知情并同意加入研究		
	排除标准	
合并严重心血管疾病患者		
疾病引起外周水肿和腹水的患者		
明确的椎管狭窄病史		
布比卡因过敏患者		
蛛网膜下腔阻滞麻醉禁忌症		
不能配合完成麻醉平面测试的患者		
正参加另一项临床试验		
与本次研究者或研究单位有利益关系者		
研究人员认为其他原因不适合临床试验者		

基本资料

姓　　名 ______________________________

身　　高 ______________________________cm

体　　重 ______________________________kg

出生年月（yyyy/mm/dd）______________

住院号码 ______________________________

手术日期（yyyy/mm/dd）______________

出院日期（yyyy/mm/dd）______________

固定电话 ______________________________

手　　机 ______________________________

联系人及电话 __________________________

邮　　编 ______________________________

家庭住址 ______________________________

手术前体格检查及辅助检查

项目	正常	异常	如异常，请详细说明	既往史
心血管（CT，MRI，和/或B超）				
肺				
胃肠道				
泌尿系统				
神经系统				
心电图				
血常规				
心肌酶谱				
肝功能				
肾功能				
电解质全套				
尿常规				
其他				

手术前合并症及药物治疗

合并症	有（具体名称）
高血压	
糖尿病	
COPD	
其他疾病	
吸烟	

围手术期相关资料

BP: mmHg; HR: 次/分; SPO_2; % ASA 分级:

手术名称:

手术用时:

宫高: 腹围: 脊柱长度:

孕次: 产次:

孕周: 胎儿数:

新生儿体重:

新生儿 1min Apgar 评分: 新生儿 5min Apgar 评分:

手术出血: ml 术中晶体: ml 术中胶体: ml

切皮前腹内压: 手术结束时腹内压:

T_4平面时间:

最高阻滞平面（温度觉消失平面）: 最高阻滞平面（痛觉消失平面）:

切皮前运动阻滞程度: 手术结束时运动阻滞程度:

术中并发症: 有/无 详情: 恶心/呕吐/寒战

血管活性药剂量

麻黄碱: mg 阿托品: mg

□ 请将复印原始检查化验报告单粘于

（2017年3月10日）

复印原始手术手术记录单粘于此页

已完成　☐ 未完成：原因________________________

请将复印原始麻醉记录单粘于此页

☐已完成　☐未完成：原因＿＿＿＿＿＿＿＿＿＿＿＿＿＿

（2017年3月10日）

主要研究者简历及参加研究人员简介

主要研究者简历

姓名：倪婷婷		性别： 女		出生年月：1983年3月	
科室	麻醉科	职称	主治医师	职务	无
教育背景(时间、院校和学位)：2003.9～2008.7 温州医学院麻醉专业获得学士学位；2011.11～2016.12 浙江大学麻醉学硕士学位。					
已发表有关临床研究的论文(题目、刊物、年、卷、页)：<br>1. 右美托咪定对体外循环下心脏手术后患者预后的影响。 中华麻醉学杂志 2016年 第36卷第7期785页-789页					
主要研究者签名：倪婷婷				日期：2017.4.10	

参加研究人员简介

姓名	职称	学历	专业	职责
潘应锋	主任医师	本科/学士	麻醉学	研究实施
贺波	主任医师	本科/学士	妇产科学	研究实施
雍安翠	副主任医师	本科/学士	妇产科学	研究实施
王璐	主治医师	本科	麻醉学	研究实施

主要研究者签名 倪婷婷　　　　日期 2017.4.10

临床研究项目负责人承诺书

本人作为　　剖宫产产妇腹内压和脊柱长度对蛛网膜下腔阻滞麻醉后感觉阻滞平面的影响　　临床研究的项目负责人向伦理委员会提出郑重声明：本人与项目申办方或资助方无不正当的利益关系，将本着实事求是、科学严谨的原则，严格按方案开展临床研究，以最高的伦理标准来保护受试者的权益和安全，确保研究结果的真实、客观、科学，承担因违反此项原则而造成的一切后果。研究结束后，积极配合国家相关监管部门 、医院等开展的项目核查工作，在接到检查通知后，认真准备核查资料，确保核查的顺利进行。

项目负责人签字：[illegible]

日期：2017 年 4 月 10 日

（2017年3月10日）

[illegible]波市第七医院临床研究者岗位职责

一、基本资料

岗位名称	临床研究者	所在部门	临床科室
岗位类别	临床研究岗位	制定日期	
任职人	[illegible]	主管部门签名	[illegible]

二、职责与工作任务描述

职责一	职责表述：职业道德规范	
	工作任务	1. 严格遵守《赫尔辛基宣言》、临床研究伦理道德和国家关于涉及人体的临床研究的相关法律法规和医院有关规定。
		2. 遵守学术道德规范，保证研究资料和数据的真实性、完整性和可追溯性。
		3. 确保本人及项目组成员与临床研究之间无经济和非经济的利益冲突，按照相关规定为研究团队进行公平合理排名。
职责二	职责表述：临床研究	
	工作任务	1. 全面负责临床研究的进行，包括研究计划的确定、执行、总结和监督。
		2. 遵守医院质量和病人安全管理的相关制度，并接受医院质量管理委员会和有关部门的监督和管理，如有不良事件和严重不良事件(SAE)发生，将按规定向上级主管部门和医院及时呈报并接受相应管理。
		3. 遵守医院关于受试者知情同意的制度和规定，保护受试者的合法权益，在研究对象的选择和研究过程中，充分告知受试者关于参加研究的风险、收益、权利、可替代方案、审批、评估和退出等的相关程序，并取得受试者的知情同意，同时保护弱势群体的利益。
		4. 为受试者保密所有研究资料和信息，必要时为上级主管机构、医院伦理委员会和相关管理部门提供资料进行审查。
		5. 按规定提供项目结题报告及年度进展报告
职责三	职责表述：团队和自我管理	
	工作任务	1. 熟悉并遵守医院各项规章制度，PI 需对研究团队的临床研究行为全面负责。
		2. 接受临床研究相关培训和继续教育，提升和完善自我素质。
		3. 接受上级主管单位、医院伦理委员会、质量管理委员会和相关管理部门监督和管理。
		4. 完成年度自我评价。
职责四	职责表述：完成上级赋予的各项临时性任务	

三、任职条件

资质	与临床相关岗位的本院员工，具有相关岗位的执业上岗资格或初级以上职称或本科以上学历；
知识技能	接受过临床研究相关培训
工作经验	具有在临床岗位、临床辅助或管理岗位工作或实习经历

本人对以上条款已阅读知晓，并承诺按要求完成职责。

任职人	签名：[illegible]	日期：2017.4.10

参考文献

[1] Ozkan ST, Orhan-Sungur M, Basaran B, et al. The effect of intra-abdominal pressure on sensory block level of single-shot spinal anesthesia for cesarean section: an observational study. Int J Obstet Anesth. 2015. 24(1): 35-40.

[2] Chun R, Baghirzada L, Tiruta C, Kirkpatrick AW. Measurement of intra-abdominal pressure in term pregnancy: a pilot study. Int J Obstet Anesth. 2012. 21(2): 135-9.

[3] Zhou QH, Zhu B, Wei CN, Yan M. Abdominal girth and vertebral column length can adjust spinal anesthesia for lower limb surgery, a prospective, observational study. BMC Anesthesiol. 2016. 16: 22.

[4] Ngaka TC, Coetzee JF, Dyer RA. The Influence of Body Mass Index on Sensorimotor Block and Vasopressor Requirement During Spinal Anesthesia for Elective Cesarean Delivery. Anesth Analg. 2016. 123(6): 1527-1534.

[5] 熊威威, 蒋奕红, 庾俊雄, 于俊芳, 赵振海. 影响剖宫产术中腰麻最高痛觉消失平面的相关因素研究. 临床合理用药杂志. 2013. 6(6): 16-17.

[6] Kirkpatrick AW, Roberts DJ, De Waele J, et al. Intra-abdominal hypertension and the abdominal compartment syndrome: updated consensus definitions and clinical practice guidelines from the World Society of the Abdominal Compartment Syndrome. Intensive Care Med. 2013. 39(7): 1190-206.
